# Discussing personalized prognosis in amyotrophic lateral sclerosis: development of a communication guide

**DOI:** 10.1186/s12883-020-02004-8

**Published:** 2020-12-14

**Authors:** Remko M. van Eenennaam, Willeke J. Kruithof, Michael A. van Es, Esther T. Kruitwagen-van Reenen, Henk-Jan Westeneng, Johanna M. A. Visser-Meily, Leonard H. van den Berg, Anita Beelen

**Affiliations:** 1 Department of Rehabilitation, Physical Therapy Science & Sports, UMC Utrecht Brain Center, University Medical Center Utrecht, Heidelberglaan 100, 3584 CX Utrecht, the Netherlands; 2 Center of Excellence for Rehabilitation Medicine, UMC Utrecht Brain Center, University Medical Center Utrecht, and De Hoogstraat Rehabilitation, Utrecht, the Netherlands; 3 Department of Neurology, UMC Utrecht Brain Center, University Medical Center Utrecht, Utrecht, the Netherlands

**Keywords:** Amyotrophic lateral sclerosis, Prognosis, Personalized prognosis, Truth disclosure, Communication guide, Physician-patient communication

## Abstract

**Background:**

*Personalized ENCALS survival prediction model* reliably estimates the personalized prognosis of patients with amyotrophic lateral sclerosis. Concerns were raised on discussing personalized prognosis without causing anxiety and destroying hope. Tailoring communication to patient readiness and patient needs mediates the impact of prognostic disclosure. We developed a communication guide to support physicians in discussing personalized prognosis tailored to individual needs and preferences of people with ALS and their families.

**Methods:**

A multidisciplinary working group of neurologists, rehabilitation physicians, and healthcare researchers A) identified relevant topics for guidance, B) conducted a systematic review on needs of patients regarding prognostic discussion in life-limiting disease, C) drafted recommendations based on evidence and expert opinion, and refined and finalized these recommendations in consensus rounds, based on feedback of an expert advisory panel (patients, family member, ethicist, and spiritual counsellor).

**Results:**

A) Topics identified for guidance were 1) filling in the *ENCALS survival model*, and interpreting outcomes and uncertainty, and 2) tailoring discussion to individual needs and preferences of patients (information needs, role and needs of family, severe cognitive impairment or frontotemporal dementia, and non-western patients). B) 17 studies were included in the systematic review. C) Consensus procedures on drafted recommendations focused on selection of outcomes, uncertainty about estimated survival, culturally sensitive communication, and lack of decisional capacity.

Recommendations for discussing the prognosis include the following: discuss prognosis based on the prognostic groups and their median survival, or, if more precise information is desired, on the interquartile range of the survival probability. Investigate needs and preferences of the patients and their families for prognostic disclosure, regardless of cultural background. If the patient does not want to know their prognosis, with patient permission discuss the prognosis with their family. If the patient is judged to lack decisional capacity, ask the family if they want to discuss the prognosis. Tailor prognostic disclosure step by step, discuss it in terms of time range, and emphasize uncertainty of individual survival time.

**Conclusion:**

This communication guide supports physicians in tailoring discussion of personalized prognosis to the individual needs and preferences of people with ALS and their families.

**Supplementary Information:**

The online version contains supplementary material available at 10.1186/s12883-020-02004-8.

## Background

Amyotrophic lateral sclerosis (ALS), also known as motor neurone disease (MND), is a neurodegenerative, incurable disease with a very heterogenous clinical presentation [1]. Life expectancy is highly variable ranging from months to over 10 years from disease onset [2]. When diagnosed with ALS, people often desire information about their prognosis [3]. Important aspects of prognosis are symptom progression (i.e. “how well”) or how their disease will affect amongst others their mobility and hand function, cognition and behaviour, and psychological symptoms, but also life expectancy (i.e. “how long”) [4]. Currently, major symptoms are discussed and patients are usually informed that the average life expectancy ranges from 3 to 5 years from disease onset. However, this covers only around 40% of people with ALS [5] and such information can result in dissatisfaction when survival falls outside this range [6]. The *Personalized European Network for the Cure of ALS (ENCALS) survival prediction model for ALS* allows a reliable estimate of survival at diagnosis (i.e. personalized prognosis); the majority of people with ALS (66%) would prefer a more personalized estimate of their life expectancy [5]. However, concerns have been raised about how to discuss the personalized prognosis in ALS appropriately and effectively without causing anxiety or destroying hope while meeting patients’ needs [7].

Communication of prognosis in a terminal disease is difficult and challenging for physicians. Unless the patient broaches the topic, physicians often do not discuss life expectancy because of physician stress, lack of training, and fear of distressing the patient and taking away hope [4, 8]. However, evidence suggests that patients can engage in prognostic discussion with minimal stress [9, 10] and are able to maintain hope by redefining what they hope for [11, 12]. Moreover, prognostic discussion may be beneficial to the patient-physician relationship [13] and patient satisfaction regarding communication [10, 14]; it may empower patients’ decision-making [12, 15, 16] and planning for the future, [15, 17, 18] and provide a sense of control [17, 19]. Avoiding the topic can have a negative impact on hope [20] and increase anxiety over time [21]. However, not all patients want to know their prognosis; the impact of prognostic discussion is mediated by patient readiness, i.e. *if* and *when* they want to know, and patient needs [11, 12, 15, 17, 19].

Breaking the news of a diagnosis of ALS is already stressful for many physicians, even experienced ones, [22, 23] something which is only compounded by the idea of also discussing personalized prognosis [7]. Prognostic disclosure, let alone that of personalized prognosis, is an underdeveloped area and important research priority in adult palliative care [4, 24]. Existing ALS guidelines offer guidance on easing the burden of the disease through symptom management, but very little support on discussing the individual life expectancy [25–27]. The aim of this study was, therefore, to develop a communication guide to support neurologists and rehabilitation physicians in discussing personalized prognosis, tailored to the individual needs and preferences of people with ALS and their families.

## Methods

A multidisciplinary working group of neurologists (MvE, HW), rehabilitation physicians (WK, EK), and healthcare researchers (RvE, AB) was formed to develop a communication guide containing recommendations on 1) using and interpreting the *ENCALS survival model* and 2) tailoring prognostic discussion to the individual needs and preferences of people with ALS and their families.

### Inventory of topics

The working group inventoried relevant topics on which guidance was needed based on reviewers’ comments on the article presenting the *ENCALS survival model* [5], feedback provided by rehabilitation physicians on presentations of the prediction model at the Dutch ALS conferences for healthcare professionals (2017, 2018), and discussions within the working group on timing, interpretation, and discussion of personalized prognosis. Furthermore, the working group selected topics for systematic review.

### Evidence on patient needs for discussing prognosis in life-limiting disease

We conducted a systematic review to determine patient needs for prognostic discussion in life-limiting disease in line with the Evidence for Policy and Practice Information (EPPI) method [28]. Review questions were formulated based on identified topics (Additional file 1. Review questions). A systematic search was conducted in MEDLINE/PubMed (up to May 2019) to find evidence (Additional file 2. Medline/PubMed search). The search was limited to original studies, systematic reviews, and patient-clinician communication guidelines. Additionally, we conducted an extended search of the references of included original studies, patient-clinician communication guidelines, [29, 30] and systematic reviews on related subjects, [8, 31–34] and a forward search using Google Scholar for articles citing included original studies. Inclusion criteria for the original studies were: full text original studies (in English) that included adult patients with a life-limiting disease receiving palliative care; investigated in-person communication between physician and patient about the life expectancy; focused on the needs of patients and their families; conducted in Europe or a western country. Findings of the studies were extracted and themes were identified based on these findings.

### Drafting the communication guide and recommendations

In the absence of evidence on discussing life expectancy in ALS, the process of formulating recommendations was based on evidence from other life-limiting diseases and expert opinion of the multidisciplinary working group. First, a subgroup (RvE, AB, WK) of the working group reviewed and discussed the evidence and drafted the initial communication guide and recommendations. Second, the guide and recommendations were discussed with the working group and finalized over two consensus meetings and one feedback round via email. In formulating the recommendations, generic communication skills such as listening, showing empathy, and checking for patient understanding were considered basic skills by the working group and, therefore, not included. Third, the guide was finalized over multiple rounds of consensus procedures together with an expert advisory panel. Because of the difficult and delicate nature of discussing life expectancy, the working group reflected on additional expertise needed and invited relevant experts to participate. Two patients with ALS and a family member (daughter) were invited as patients and caregivers representatives. An ethicist was consulted to support in tailoring discussion of life expectancy in a manner that is respectful of the needs of individual patients and their families. A spiritual counsellor with an Islamic background was invited to ensure that recommendations match the needs of patients with a different cultural background; in the Netherlands, spiritual counsellors provide support and reflect on beliefs and values of patients and their family regardless of their faith or belief system. An independent rehabilitation physician not connected to the ALS Center Netherlands was invited to review the communication guide from the perspective of rehabilitation physicians who coordinate the multidisciplinary care for patients after the diagnosis. The expert advisory panel reviewed the guide and provided feedback via email; their feedback was discussed by the working group via email and used to further refine the guide. This process was repeated until the expert panel reached consensus.

## Results

### Topics for guidance

Identified topics were divided over two categories. 1) Using and interpreting the *ENCALS survival model*: a) filling in the model and dealing with missing, incorrect or unclear values; b) selecting and interpreting the outcomes; c) communicating the results to the patient; d) uncertainty in estimates of survival; e) timing of prognostic discussion. 2) Individual needs and preferences of people with ALS and their families: a) information needs patient; b) role and needs family; c) patients with severe cognitive impairment and frontotemporal dementia (FTD); d) immigrant patients with a non-western background in the Netherlands.

### Evidence on patient needs for discussing prognosis in life-limiting disease

**Fig. 1:**
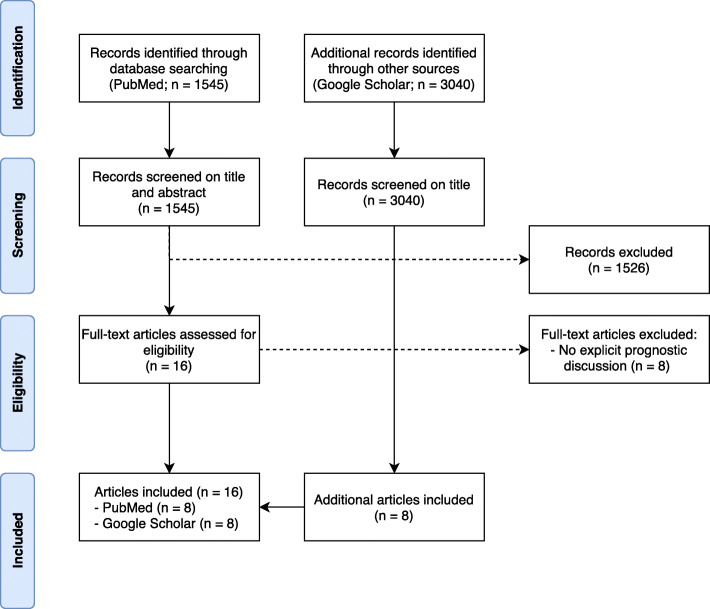
Prisma flow chart of inclusion studies

A total of 17 studies were included in the review (Fig. 1). Two studies provided evidence on patients with an immigrant background in the Netherlands, 15 studies provided evidence on other patient needs. An additional file contains study characteristics, study findings, synthesis of findings, and references of included studies (Additional files 3, 4, 5 and 6). 15 of the 17 studies focused exclusively on patients with advanced, incurable cancer; none of the studies included patients with a neurodegenerative disease.

Based on a synthesis of the evidence, the following themes were identified: tailored information, family support, diverging information needs, and conspiracy of silence. No evidence was found on tailoring discussion to patients with severe cognitive impairments or FTD.

i. *Information needs patient*. Tailored information. Not all patients want to know their prognosis [18, 19, 35–37]. Information needs differ from patient to patient and prognostic disclosure should be tailored to individual needs [15–20, 35–37]. Asking how much patients want to know, without explaining what information is available and exploring their emotions and concerns, might not be sufficient to elicit their need for information [15, 19, 37]. Some patients want more explicit prognostic information and time frames, whereas others desire a more general indication [17, 19, 35–37]. Some expressed the hope of being on the tail of the (survival) curve, [11, 35] whereas others did not want to hear statistics and time frames fearing that these could potentially cause them distress and threaten their hope [15, 18]. Although patients emphasized that false hope should not be encouraged and uncertainty should be underlined, some patients emphasized the need for physicians to provide hope by indicating positive aspects and good news stories about other patients beating the odds [11, 17, 21, 35]. Furthermore, patients emphasized that physicians should explain that statistics are group estimates which may not apply to the individual [8, 18].
ii. *Role and needs family*. Family support. Most patients want to have family members present to provide emotional support during prognostic discussion, but patients said this should be the patient’s choice [16, 17, 36, 38]. Diverging information needs. The families’ needs for information can diverge from those of the patient [16, 19, 35, 37, 38]. Even if a patient does not want to know their prognosis, it is possible that their family does want this information which can help them plan for the future and care requirements [15, 16, 38]. In this case, according to patients, the prognosis can be discussed with their family if they want to know and provided the patient has given permission [19, 37, 38]. Although patients and their families might wish to protect each other from bad news, families respected the patients’ right and wish to know [16, 18].
iii. *Non-western patients with an immigrant background in the Netherlands*. Conspiracy of silence. Families of immigrant patients in the Netherlands, specifically Muslim patients, may prefer to function as an intermediate in prognostic discussion [39–41]. This can result in them maintaining a conspiracy of silence in order to protect the patients’ hope and because of different values and beliefs related to health and dying [39–41]. This can create tensions between the values of Dutch healthcare providers desiring open discussion with the patient [39]. However, this difference in values and the topic of life expectancy can be discussed if done in a culturally sensitive manner [39–41].

### Communication guide and consensus procedures

Our recommendations in the communication guide have been divided into three parts (Table 1). The first part deals with practical aspects of filling in and interpreting the prediction model, how to deal with missing or incomplete data, uncertainties of the model and estimated survival, how to interpret the results, and which outcomes of the *ENCALS survival model* to discuss. The second part covers tailoring prognostic discussion to the needs and preferences of individual patients and their families. The third part contains tips on how to provide information on individual life expectancy in stepwise fashion tailored to patient preference, starting with the situation in general (i.e. prognostic groups), and then, if preferred, addressing more specific points (i.e. interquartile range (IQR) of the survival probability).

**Table 1:** Overview of recommendations for discussing personalized prognosis with people with ALS and their families

**1. Interpreting the ENCALS survival model**	
The ENCALS survival model provides three outcomes: 1) survival curve; 2) risk group (i.e. very short, short, intermediate, long , or very long); 3) survival probability and interquartile range.	
1. Do not use the survival curve to discuss personalized prognosis, this may overwhelm the patient.	
2. Discuss the personalized prognosis based on the risk group, the group median, or the interquartile range of the survival probability (see 3.1 below).	
**2. Tailoring discussion to individual patient needs**	
*2.1 General*	
1. Tailor discussion of personalized prognosis to patient readiness and individual information needs.	
2. The patient has a right not to know their prognosis.	
*2.2 Family and next of kin*	
1. Stimulate patients to bring family or next of kin with them for support.	
2. If the patient requests it, discuss their prognosis first with their family or next of kin.	
*2.3 Diverging information needs*	
1. If the patient does not want to know their prognosis, but their family or next of kin does, only discuss prognosis with family or next of kin after obtaining the patient’s permission.	
*2.4 Non-western patients with an immigrant background in the Netherlands*	
1. If there is a language barrier, use a professional translator.	
2. Similar to all patients, explore the needs and preferences of patients with a different cultural background, and their families or next of kin, with regard to discussing their prognosis.	
3. Family or next of kin of non-western patients might try to shield the patient from their prognosis. If the patient requests it, discuss their prognosis with their family or next of kin.	
*2.5 Patients with serious cognitive impairments/FTD*	
1. If due to cognitive impairment/FTD the patient is suspected of lacking decisional capacity to decide whether they want to discuss their prognosis, a cognitive screener like the Edinburgh Cognitive and Behavioral ALS Screen can be used to gain insight into affected cognitive domains.	
2. If the patient is judged to lack decisional capacity to decide whether they want to discuss their prognosis, ask their family or next of kin if they want information about the prognosis.	
**3. Discussing personalized prognosis**	
*3.1 General*	
1. Ask the patient how much they would like to know and tailor discussion to their preferences.	
2. Differentiate between three steps of increasing detail	
i. **Risk groups** without a time indication: very short, short, intermediate, long, or very long.	
ii. **Group average** as a time indication: very short (1.5 years), short (2 years), intermediate (3 years), long (3.5 years), or very long (7.5 years).	
iii. **Interquartile range** of the survival probability if the patient requests a more individual estimation of their prognosis.	
3. Emphasize that the prognosis is not an exact time frame, but an estimation and that individual disease progression varies per patient. Point out the long tail (on the graph) and explain that half of the patients live longer, some of whom much longer.	
*3.2 Example prognostic discussion*	
1. **Risk group:**	
◌“Looking at your disease characteristics, you fall into the group with a [much shorter than average / shorter than average / intermediate / longer than average / much longer than average] life expectancy.”	
◌“Half of the patients in every group live longer than the average, some of whom much longer.”	
2. **Group average:**	
◌“In this group, half of the people die within the first [1.5 years (much shorter) / 2 years (shorter) / 3 years (average) / 3.5 years (long) / 7.5 years (much longer)] of their disease.”	
◌“The other half live longer, some of whom much longer.”	
3. **Interquartile range**	
◌“Of the patients with your disease characteristics, two out of four die between … months [75^th^ percentile] and … months [25^th^ percentile].”	
◌“However, one in four patients dies earlier, but one in four lives longer, some of whom much longer.”	

During the consensus procedures the following topics were discussed between the working group and expert panel. First, selection of outcomes of the *ENCALS survival model* (i.e. prognostic groups, survival curve, survival probability) to discuss and illustrate the estimated life expectancy. Initially, we only considered the prognostic groups and their median ranges to be suitable for this purpose. We assumed that survival curves and survival probability might overwhelm a patient. However, after exploratory discussions of prognosis by members of the working group (MvE, HW, EK), we concluded the IQR of the survival probability to be suitable for illustrating a more individualized estimation. Second, uncertainty in estimates of survival. We recommended that uncertainty of individual disease progress be emphasized by discussing life expectancy as a group range while also pointing out that some patients within this group are better off and others worse off. This can be further illustrated using the interquartile range of the survival probability. Third, timing of prognostic discussion. The working group deliberated whether personalized prognosis could be discussed during diagnosis given the limited time available to fill in the prediction model during consultation, and whether patients would be able to process the information considering the emotional impact of the diagnosis. Due to a lack of evidence, we decided not to make a recommendation on the preferred timing. However, we concluded it would be unethical to continue telling them the average life expectancy without mentioning the possibility of a more personalized prognosis; the option to discuss personalized prognosis, if the patient wants to know, should be offered during diagnosis. Fourth, recommendation on culturally sensitive communication. The working group concluded that offering spiritual assistance while discussing the prognosis is part of core patient-centered communication skills and recommendations on this were not included. We did include recommendations on how to discuss personalized prognosis in a culturally sensitive manner. Fifth, lack of disease insight versus lack of decisional capacity in cognitively impaired patients. The ethicist in our expert panel suggested we should make a more clear distinction between these, since the latter comes with certain patient rights and physician responsibilities. To avoid ambiguity, the working group decided to focus our recommendations specifically on patients lacking decisional capacity to decide whether they want to discuss their life expectancy. An additional recommendation was to use a cognitive screener like the Edinburgh Cognitive and Behavioral ALS Screen (ECAS) to gain insight into affected domains if a lack of decisional capacity is suspected. Finally, the working group discussed whether percentages (50% of patients) or frequencies (2 out 4 patients) should be used to discuss the IQR. We concluded that patients are more likely to understand survival if expressed as a frequency.

In addition to the consensus procedures, a preliminary version of the guide was discussed with rehabilitation physicians working in ALS care during a workshop at the Dutch ALS conference for health professionals (2019). Their comments on filling in the prediction model (including ‘conversion’ of progressive muscular atrophy (PMA) or primary lateral sclerosis(PLS) to ALS, patient’s country of origin, forced vital capacity upright or supine, and using the model to track disease progression) were incorporated in the text.

## Discussion

We have developed a communication guide to support physicians in discussing personalized prognosis in ALS. Recommendations aim to provide guidance in filling in and interpreting the *ENCALS survival model* and support physicians in tailoring discussion of personalized prognosis to the individual preferences and needs of people with ALS and their families. Uncertainty in estimation of life expectancy, due to heterogenous individual disease progression as well as inherent limitations of the underlying prediction model, are discussed [5]. Finally, patient choice and the right not to know are emphasized as the basis for prognostic discussion.

### Communication of personalized prognosis

Our communication guide focuses on discussing estimated life expectancy based on the *ENCALS survival model*. Discussion of life expectancy (i.e. quantity) can support the quality of life of patients by aiding patients and their families in decision-making [12, 15, 16] and planning for their care and future [15, 17, 18], as well as providing patients a sense of control [17, 19]. It can also support healthcare professionals in the timing of appropriate and effective care easing the burden of the disease [25]. However, how to provide numerical estimates of survival and associated uncertainties in a manner that supports patient decision-making is a subject of debate [42, 43]. Being too specific can cause distress if survival is underestimated or overestimated, [6] but too wide a range can reduce credibility and accurate understanding [44]. It has, therefore, been argued in oncology and neurology that life expectancy can be discussed effectively using multiple scenarios based on the median and interquartile range to illustrate average survival, and groups worse and better off [4, 45]. This can also help patients prepare for the worst while hoping for the best; a study in cancer patients showed that patients preferred this to simply median survival [46]. Another possible barrier to patient understanding is statistical illiteracy [47]. Visual aids can help facilitate patient understanding, [48] but patients generally prefer words and numbers to graphs and diagrams [49, 50]. Whether estimated survival is communicated visually or in words and numbers, patient understanding can be supported using frequencies instead of single events, absolute rather than relative risk, mortality not survival, and natural frequencies rather than conditional probabilities [47, 51] as we have done in our recommendations.

### Non-western patients with an immigrant background

Studies amongst general practitioners and oncologists show that physicians often communicate differently with non-western patients with an immigrant background: consultations are shorter and less focused on involvement and empathy, [52] patients are involved less in decision-making, [53] and more medical jargon is used [54]. However, it is not at all evident that patient needs for prognostic discussion differ between western and non-western patients. Some, but not all, want to know their life expectancy, [18] desire the topic to be discussed first or only with their family, [18, 39, 41] and prefer a more indirect style of communication [39, 41]. Thus, many core skills of patient-centered communication are relevant during intercultural communication [55]. However, one important difference is the role of family. Families of western patients emphasize the importance of respecting the patient’s choice in knowing their prognosis, even though sometimes they would prefer to protect the patient from bad news [16, 18]. Whereas families of non-western patients often prefer to shield the patient from bad news, in order to protect their hope [18, 39, 41]. However, western healthcare values and laws respect patients’ autonomy, including the choice of not wanting to know or letting family make this decision.

### Impact of cognitive impairments in discussing prognosis

Cognitive or behavioral changes occur in up to half the patients with ALS, [1] which can impact patient autonomy in, amongst others, decision-making and communication of personalized prognosis. Around 13% of patients with ALS fulfill the criteria for the behavioral variant of FTD, [1, 56] which can cause apathy, reduce insight, and impair decision-making [57]. However, this does not necessarily mean the patient lacks decisional capacity. Therefore, the working group decided to differentiate between cognitive impairment versus a lack of decisional capacity regarding decision-making on discussion of life, and focus our recommendations on the latter. If a lack of decisional capacity is suspected, a cognitive screener can be used to provide insight into affected cognitive domains. A concise screener like the ALS-CBS could be used to screen for behavioral changes; however, a broader screener like the ECAS is recommended because difficulties in decision-making can also be caused by other domains like impaired language or memory [58]. Assessing decisional capacity depends on the physician’s judgement and weighing of multiple relevant factors in addition to cognition (e.g. emotion, motivation, and volition), is specific to the situation, and subject to different legal definitions depending on the country [57]. Discussing estimated survival with the patient’s family, if they want to know, can still be important as they will have to take into account a poorer prognosis due to cognitive impairment [5, 59].

### Generalizability

When using the *ENCALS survival model,* two limitations have to be taken into consideration. First, although it is becoming more common to consider ALS, PMA, and PLS to be on a spectrum within the same disease, [1] the model has only been validated in patients with ALS [5]. Second, the model has been developed and calibrated with data from 14 ALS centers across 9 countries [5] and can be used to reliably estimate prognosis for these countries using their cohort. Other western countries can use the general ENCALS survival model which can be tailored to regional factors by recalibration of the intercept of the prediction model in future studies. However, the model has not been calibrated for countries in Asia, South-America or Africa, and differences in genetics, healthcare systems, and other factors have thus not been taken into account. An additional consideration is that this guide was developed in the Dutch healthcare setting. However, we believe that our recommendations can be useful to support discussion of personalized prognosis in other western countries. Evidence underlying recommendations, except those on immigrant patients in the Netherlands, comes from international studies and are in line with international guidelines on communication in cancer [29, 30].

### Specificity

While conducting our review, we found no evidence on discussing life expectancy in ALS. Available evidence was mainly based on patients and family caregivers in terminal cancer. It is unclear whether these findings can be generalized to ALS. Whereas in most cancers people are able to retain some hope of being cured, the disease outcome in ALS is homogenous in its invariable lethality and relentless, unavoidable and constant prospect of decline and loss [60]. Possibly as a result, patients with ALS more often engage in advance care planning compared to those with cancer, [61] which can necessitate more information on personalized prognosis. On the other hand, cognitive impairment plays a much more significant role in ALS, even early in disease, [62] which can hinder decision-making and impact decisional capacity, [57] a topic absent from patient-clinician communication guidelines in cancer [29, 30].

### Strengths

This is the first communication guide, as far as we are aware, on tailoring discussion of personalized prognosis in life-limiting disease based on a prediction model. Additional strengths of this project are inventory of topics amongst the target audience, development over multiple rounds of consensus procedures, and feedback by a broad expert panel which included people with ALS and a family member.

### Limitations

One limitation of our guide is that the underlying evidence was obtained from studies in patients with terminal cancer and this may not be valid for patients with ALS. A second limitation concerns our search to identify the needs of immigrant patients with a non-western background in the Netherlands. We only found evidence on the needs of Muslim patients with a predominantly Turkish or Moroccan background, [41, 63] the two largest immigrant groups in the Netherlands [64]. However, in formulating our recommendations, the working group and expert panel did take into account all immigrant groups in the Netherlands and our recommendations are in line with Dutch consensus recommendations on palliative care for people with an immigrant background [65].

### Implementation

The *ENCALS survival model* is accessible to physicians and researchers by registering online [66]. This communication guide is intended to facilitate discussion of personalized prognosis in ALS and will be distributed through the network of the ALS Center Netherlands. In addition, the full Dutch version and an abbreviated English version will be made available online at our website [67]. However, the development of this communication guide is only the first step in the implementation of discussion of personalized prognosis. We are currently conducting a qualitative study to evaluate patient and caregiver experiences with discussing personalized prognosis based on our communication guide. The results of this study will be used to provide recommendations on discussing life expectancy in ALS and the guide will be adapted accordingly.

## Conclusion

This communication guide supports physicians in filling in and interpreting the *ENCALS survival model* while tailoring discussion of personalized prognosis to the individual needs and preferences of people with ALS and their families. Uncertainty of estimated survival and individual disease progression should be emphasized by discussing the estimated life expectancy as a range and underlining that some patients are better off and some worse off. Prognostic discussion should be tailored to individual information needs and preferred level of explicitness. Patients should be given the choice of having family present for emotional support. Families of patients with a non-western background may try to shield the patient from bad news about their prognosis, but, while respecting cultural values, physicians should explain that this is the patient’s choice. When information needs diverge and the patient does not want to know their prognosis, this can be discussed with the family with patient permission. Whether to discuss personalized prognosis or not is always the choice of the patient, including the right not to know. However, if the physician judges that the patient lacks the capacity to make this decision due to severe cognitive impairments or FTD, an exception should be made and life expectancy discussed with their family. An ongoing, qualitative study is currently evaluating the effect of tailored discussion of personalized prognosis on patients with ALS.

## Supplementary Information

**Additional file 1.** Review questions.

**Additional file 2.** MEDLINE_PubMed search.

**Additional file 3.** Table 1. Study characteristics.

**Additional file 4.** Table 2. Study findings.

**Additional file 5.** Table 3. Synthesis of findings.

**Additional file 6.** Full list of included studies.

## Data Availability

The articles in this study are available via MEDLINE/PubMed and Google Scholar. All data from the systematic review are available in the appendix.

## Abbreviations

ALS: Amyotrophic Lateral Sclerosis
MND: Motor Neurone Disease
ENCALS: European Network for the Cure of ALS
FTD: Frontotemporal dementia
IQR: Interquartile range
PMA: Progressive Muscular Atrophy
PLS: Primary Lateral Sclerosis
ECAS: Edinburgh Cognitive and Behavioral ALS Screen
