# Supplementary material for: Discussing personalized prognosis in amyotrophic lateral sclerosis: development of a communication guide
Source: BMC Neurol. 2020 Dec 14;20:446. doi: 10.1186/s12883-020-02004-8 (PMC7734773; doi:10.1186/s12883-020-02004-8)
Supplement: Supplementary file 1 — Additional file 1. Review questions. [file 12883_2020_2004_MOESM1_ESM.docx]

**Review questions**

1. What are patient needs for discussing prognosis in a life-limiting disease?
   1. What are information needs of patients when discussing prognosis?
   2. What is the role and what are information needs of caregivers/family when discussing prognosis?
   3. What are specific needs of patients with serious cognitive impairments or ALS-FTD?
2. What are specific needs of non-Western patients in the Netherlands?
