# Supplementary material for: Discussing personalized prognosis in amyotrophic lateral sclerosis: development of a communication guide
Source: BMC Neurol. 2020 Dec 14;20:446. doi: 10.1186/s12883-020-02004-8 (PMC7734773; doi:10.1186/s12883-020-02004-8)
Supplement: Supplementary file 2 — Additional file 2. MEDLINE_PubMed search. [file 12883_2020_2004_MOESM2_ESM.docx]

**MEDLINE/PubMed search**

**Database**: MEDLINE (PubMed).

**Search (research question 1)**:

("communication"[MeSH Major Topic] OR "communication"[Title/Abstract] OR "conversation"[Title/Abstract] OR "Physician-Patient Relations"[MeSH Major Topic] OR "Physician-Patient relations"[Title/Abstract] OR "patient physician relationship"[Title/Abstract] OR "physician patient relationship"[Title/Abstract] OR "patient professional relationship"[Title/Abstract] OR "professional patient relationship"[Title/Abstract] OR ("patient"[Title/Abstract] AND ("physician"[Title/Abstract] OR "professional"[Title/Abstract]) AND "relationship"[Title/Abstract])) AND ("attitude to death"[MeSH Major Topic] OR "attitude to death"[Title/Abstract] OR ("attitude"[Title/Abstract] AND "death"[Title/Abstract]) OR "prognosis"[MeSH Major Topic] OR "prognosis"[Title/Abstract] OR "life expectancy"[MeSH Major Topic] OR "life expectancy"[Title/Abstract] OR ("expectancy"[Title/Abstract] AND "life"[Title/Abstract]) OR "truth disclosure"[MeSH Major Topic] OR "truth disclosure"[Title/Abstract] OR ("disclosure"[Title/Abstract] AND "truth"[Title/Abstract]) OR "prognostic disclosure"[Title/Abstract] OR ("prognostic"[Title/Abstract] AND "disclosure"[Title/Abstract])) AND ("terminal care"[MeSH Major Topic] OR "terminal care"[Title/Abstract] OR ("terminal"[Title/Abstract] AND "care"[Title/Abstract]) OR "palliative care"[MeSH Major Topic] OR "palliative care"[Title/Abstract] OR ("palliative"[Title/Abstract] AND "care"[Title/Abstract]) OR "end-of-life"[All Fields])

**Total hits PubMed:** 1545.

**Search date**: 03-05-2019.

**Inclusion criteria**: full text original studies (in English) that included adult patients with a life-limiting disease receiving palliative care; investigated in-person communication between physician and patient about the life expectancy; focused on the needs of patients and their families; conducted in Europe or a Western country.

**Search (research question 2)**:

("communication"[MeSH Major Topic] OR "communication"[Title/Abstract] OR "conversation"[Title/Abstract] OR "Physician-Patient Relations"[MeSH Major Topic] OR "Physician-Patient relations"[Title/Abstract] OR "patient physician relationship"[Title/Abstract] OR "physician patient relationship"[Title/Abstract] OR "patient professional relationship"[Title/Abstract] OR "professional patient relationship"[Title/Abstract] OR ("patient"[Title/Abstract] AND ("physician"[Title/Abstract] OR "professional"[Title/Abstract]) AND "relationship"[Title/Abstract])) AND ("terminal care"[MeSH Major Topic] OR "terminal care"[Title/Abstract] OR ("terminal"[Title/Abstract] AND "care"[Title/Abstract]) OR "attitude to death"[MeSH Major Topic] OR "attitude to death"[Title/Abstract] OR ("attitude"[Title/Abstract] AND "death"[Title/Abstract]) OR "prognosis"[MeSH Major Topic] OR "prognosis"[Title/Abstract] OR "life expectancy"[MeSH Major Topic] OR "life expectancy"[Title/Abstract] OR ("expectancy"[Title/Abstract] AND "life"[Title/Abstract]) OR "truth disclosure"[MeSH Major Topic] OR "truth disclosure"[Title/Abstract] OR ("disclosure"[Title/Abstract] AND "truth"[Title/Abstract]) OR "prognostic disclosure"[Title/Abstract] OR ("prognostic"[Title/Abstract] AND "disclosure"[Title/Abstract])) AND ("cultural diversity"[MeSH Major Topic] OR "cultural diversity"[Title/Abstract] OR ("cultural"[Title/Abstract] AND "diversity"[Title/Abstract]) OR "Transients and Migrants"[MeSH Major Topic] OR "Transients and Migrants"[Title/Abstract] OR ("Migrants"[Title/Abstract] AND "Transients"[Title/Abstract]) OR "cultural competency"[MeSH Major Topic] OR "cultural competence"[Title/Abstract] OR "cultural competency"[Title/Abstract] OR "culturally competent care"[MeSH Major Topic] OR "culturally competent care"[Title/Abstract] OR ("culturally"[Title/Abstract] AND "competent"[Title/Abstract] AND "care"[Title/Abstract]) OR "Emigrants and Immigrants"[MeSH Major Topic] OR "Minority Groups"[MeSH Major Topic] OR "Islam"[MeSH Major Topic] OR "Islam"[Title/Abstract])

**Total hits PubMed**: 154.

**Search date**: 03-05-2019.

**Inclusion criteria**: full text papers (in English) on original studies that included adult patients with a life-limiting disease receiving palliative care; investigated in-person communication between physician and patient about the life expectancy; focused on the needs of patients and their caregivers; focused on the needs of patients and their caregivers with a non-Western background in the Netherlands.
