# Supplementary material for: Discussing personalized prognosis in amyotrophic lateral sclerosis: development of a communication guide
Source: BMC Neurol. 2020 Dec 14;20:446. doi: 10.1186/s12883-020-02004-8 (PMC7734773; doi:10.1186/s12883-020-02004-8)
Supplement: Supplementary file 3 — Additional file 3. Table 1. Study characteristics. [file 12883_2020_2004_MOESM3_ESM.docx]

Table 1. Study characteristics

| **Nr.** | **Study** | **Study purpose** | **Design** | **Methods** | **Sample /setting** |
| --- | --- | --- | --- | --- | --- |
| [1] | Butow 2002 | To obtain patient and health professional views on optimal ways of presenting prognosis to patients with metastatic cancer. | Qualitative study. | Semi-structured interviews with detailed probes. | *N* = 17; women with metastatic breast cancer. Australia |
| [2–5] | Clayton 2005 † | To examine the views of terminally ill patients, caregivers, and PC HPs on fostering coping and hope, preferred content of information for discussion of life expectancy and by whom, how, and when the discussion should be conducted. | Qualitative study. | Focus groups and individual interviews with patients unable to attend the focus groups. | *N* = 19; palliative care patients (advanced cancer). Australia. |
| [6] | Coulourides Kogan 2015 | To explore seriously ill patients perspective and experience of an IPC consultation, and to explore patient attitudes toward the information derived from the consultation. | Qualitative study | Semi-structured interviews the week after IPC (Initial Palliative Care) consultation. | N=11; terminally ill patients in palliative care. USA |
| [7] | Curtis 2008 | To study the interactions between the desire to have hope supported and need to receive explicit prognostic information amongst patients, family and HCP. | Qualitative study. | Semi-structured interviews. | *N* = 55; Patients with advanced cancer (30) and severe COPD (24), or both (1). USA |
| [8–10] | De Graaff 2010-2012 †† | To explore how Dutch professional care providers deal with Turkish and Moroccan immigrants ideas on palliative care, what influences communication and decision-making, and the influence of different styles of care management. | Qualitative study. | Semi-structured interviews. | *N=* 36; Turkish or Moroccan patients with incurable cancer (6) and relatives (30). The Netherlands |
| [11] | Friedrichsen 2011 | To explore the experiences and preferences of terminally ill cancer patients regarding truth telling in the communication of poor prognoses. | Qualitative study. | Semi-structured interviews. | *N* = 45; terminally ill cancer patients in palliative care. Sweden |
| [12] | Hagerty 2005 | To identify preferences for the process of prognostic discussion among patients with incurable metastatic cancer. | Observational study. | Postal survey measuring patient preferences for manner of delivery of prognostic information, including how doctors might instill hope. | *N* = 126; patients with metastatic cancer. Australia |
| [13] | Kirk 2004 | To elicit views of patients in palliative care and their family members regarding their experiences of information disclosure about the illness. | Qualitative study | Semi structured interviews of participants’ perceptions of their experiences of disclosure about the illness. | *N* = 37; patients in palliative cancer care in Australia (*N* = 21) and Canada (*N* = 16). |
| [14] | Mitchison 2012 | To explore personal experiences of and preferences for prognostic communication in migrant and Anglo-Australian patients. | Qualitative study | Structured interviews. | *N* = 31; Anglo-Australian patients with metastatic cancer and 25 family members; responses of immigrant groups were not included. Australia |
| [15] | Oosterveld-Vlug 2017 | To explore how Dutch patients and relatives with and without a Muslim background think that realistic and hopeful information should be combined in physician–patient communication at the end of life. | Qualitative study | Online focus group. | *N* = 9; patients and relatives with a Muslim background. The Netherlands. |
| [16] | Rohde 2019 | To explore experiences of patients with incurable colorectal cancer while in palliative care and their reflections on the information provided, specifically disease, prognosis and life expectancy. | Qualitative study. | Semi-structured interviews. | *N* = 20; patients with colorectal cancer receiving palliative chemotherapy. Norway |
| [17] | Walczak 2013 | To explore patients perspectives across two cultures (Australia and USA) regarding optimal communication about prognosis and end-of-life care issues. | Qualitative study. | Semi-structured individual interviews and focus group. | *N* = 34; patients with advanced, incurable cancer. Australia (*N* = 15) and USA (*N*  = 19) |

† The results of this study were split over four separate articles.

†† The results of this study were split over three separate articles.
