# Supplementary material for: Discussing personalized prognosis in amyotrophic lateral sclerosis: development of a communication guide
Source: BMC Neurol. 2020 Dec 14;20:446. doi: 10.1186/s12883-020-02004-8 (PMC7734773; doi:10.1186/s12883-020-02004-8)
Supplement: Supplementary file 4 — Additional file 4. Table 2. Study findings. [file 12883_2020_2004_MOESM4_ESM.docx]

Table 2. Study findings

| **Nr** | **Study** | **Results** | **Discussion & Conclusions** |
| --- | --- | --- | --- |
| [1] | Butow 2002 | 1. Patient needs  *Communication within a caring, trusting, long-term relationship:* Patients express the view that they wanted to hear their prognosis from their oncologist, whom they knew and trusted.  *Clear, straight-forward presentation of prognosis where desired:* Most women wanted prognostic information to be disclosed in a straightforward, honest manner, if desired. This method of disclosure was seen to have several positive outcomes, including reassurance, promotion of trust and good coping, effective decision-making and planning, and protection against false expectations.  *Encouragement of hope and a sense of control:* Hope was mentioned by all the patients as a vital part of prognostic discussions. They saw this as a distinguishing feature of the “good” doctor.  1a. Information needs  *Strategies to ensure patient understanding:* Some women also emphasised the need for doctors to assess whether the patient is capable of comprehending the statistics presented, and more importantly, whether they can interpolate these figures to their own situation.  Most patients wanted an honest appraisal of their situation, but were wary of statistics, especially a time frame. They saw statistics as potentially hope-destroying and wanted to hear “good news” stories. | 1. Patient needs  All interviewees emphasised the importance of conveying hope. All respondents indicated the need for realism and honesty to temper hope-giving, but felt that this could be achieved even within the most hopeless scenario.  1a. Information needs  Participants felt that the health professional needed to carefully explore with the patient what information they want, how they will use such information and how such information might most usefully be imparted to them. The end result of such a discussion may be far from the presentation of a survival curve. |
| [2] | Clayton 2005a | 1. Patient needs  All participant groups said that the manner in which the information is given is often more important than what is actually said.  1a. Information needs  *General indication, not a time frame:* Many patients and carers said they did not want to be given a time frame, but wanted a general indication of what to expect in the future.  *A time frame if requested:* Some patients and carers said that it was important to them to be given a survival time frame. A few patients and carers expressed frustration that they had not been given this information.  *Avoid being too exact:* Some patients stressed that it is important not to be too restrictive or definite with time frames, because patients may fixate on this.  *Various ways to phrase time frames:* Those patients and carers who wanted to be given a time frame mostly said they would like to know how long the average person with their condition would live and/or be given a rough range. A few said they would like to know the longest possible time that they might live.  *Statistics:* Patients and carers also said that it is important to explain that statistics apply to a group so they can only be used as a guide. Patients and carers wanted their HPs to highlight that every person is an individual and that people’s experiences are different even with the same disease.  1b. Role and needs family  Some carers said their reasons for needing a time frame were different to those of the patient; for example, knowing how much time to take off work and whether to call other family members to share the care-giving burden. | 1a. Information needs  While most patients said it was very important to be informed that their illness would limit their lifespan, not all wanted to be told detailed information about their life expectancy. |
| [3] | Clayton 2005b | 1. Patient needs  All participants groups believed that there were ways of fostering coping and nurturing hope when discussing prognosis and EOL issues with terminally ill cancer patients and their caregivers.  **Emphasize what can be done**  *Control of physical symptoms:* Patients said that it is important to reassure patients that pain and other symptoms can be controlled.  *Emotional support, care, and dignity*: Patients said that it is vital for the HP to convey the sense that they care about the patient and to show compassion. The value of listening and acknowledging the emotional concerns of the individuals involved also was highlighted. Patients emphasized that patients need to know that their physician and other HPs are doing their utmost to help them, and that they will not be abandoned, they will have plenty of support.  *Practical support*: Patients commented that it was reassuring to be informed about equipment and resources that are available.  **Balance between truth and hope**  Several patients said that it is important to be honest with patients when discussing the future. None of the patients and caregivers indicated that they did not want their HP to be honest. Some participants even said that it gave them hope when the HP was honest. Nevertheless, patients … stated that it is important not to be too blunt or provide a great deal of detailed information that the patient does not want to hear.  The need to maintain hope was emphasized by all the participant groups. Some patients and caregivers said that it is important for HPs not to give false hope but any positive aspects should be emphasized.  **The spectrum of hope**  Several patients and some caregivers also spoke about a range of ways to find hope in their situation. The hope of being well cared for and supported by HPs was the source of hope that was mentioned most frequently by patients and caregivers. A few patients spoke of the hope of beating the odds and being on the tail of the survival curve. The inaccuracy and uncertainty of the prediction of life expectancy were seen as potential causes for hope because the person may live longer than average. | 1. Patient needs  The value of emphasizing what can be done in terms of the control of physical symptoms; emotional support, care, and dignity; and practical support was highlighted by all participant groups.  The importance of being honest while at the same time not imposing the truth about a patient’s prognosis when it was not wanted was emphasized. Similarly, pointing out the positive aspects while not encouraging the patient’s false hopes also was raised by all participant groups. |
| [4] | Clayton 2005c | 1. Patient needs  **Who and When to Initiate Discussions About Prognosis and Eol issues**  *Wait for the patient to raise the topic:* Some patients felt it should be up to patient and/or carer to initiate the discussion. A few patients and carers spoke of the patients’ right to be protected and not have painful discussions about prognosis.  *Offer all patients the opportunity to discuss the future*: Most patients and carers said they thought it would be alright for the doctor to offer to discuss prognosis provided they had the option of saying they did not want this information. Of note, no patients, carers, or HPs felt that the doctor or nurse should bring up the facts out of the blue without checking first whether the patient or carer wanted this information, as it was felt to be important to respect people’s right not to know.  *Initiate the discussion when the patient seem ready*: Some patients said that HPs should initiate the discussion when they think the patient is ready.  **Optimal context**  *Relationship with the health professional*: Patients strongly emphasized the importance of being comfortable with their HP when discussing prognosis. They said that it is vital for the HP to show compassion and respect and to ensure that adequate support is present. Some patients spoke of the devastating effect of having bad news broken when the doctor did not show any signs of compassion.  *Negotiate who should deliver the information:* Most patients and carers, if they wanted to have the discussion at all, wanted to discuss prognosis and EOL issues with a doctor or nurse. However, one carer said her husband wanted their priest and not the doctor to deliver any bad news and had requested this be documented in the medical record. Another patient requested that the family be told first and for the family, not the doctor, to be the one to tell him.  1a. Information needs  *Clarify how much the patient wants to know:* All patients said that it is important to tailor the information to the individual patients’ preference and be aware that this may change over time.  1b. Role and needs family  *Negotiate who should be present during the discussion:* Most patients wanted someone from their immediate family present but one patient was angry when her initial diagnosis was disclosed in front of her son, as she felt she should be the one to tell him. Some patients said they valued being able to discuss sensitive topics, such as dying, on their own with a PC HP, because they did not want to worry their family about these issues. In general, participants felt that it was important to negotiate who should be present when bad news was given. | 1. Patient needs  Provided the patient and/or carer is given the option not to hear the prognosis and discuss EOL issues and the topic is broached in a sensitive manner, most participants felt that it was appropriate and important for the doctor or nurse to make this an accessible topic, because the patient might find it difficult to raise it themselves.  1a. Information needs  Patients varied in the amount of information that they would want regarding prognosis and EOL issues. Together these findings stress the importance of clarifying with patients how much detail they want to know.  1b. Role and needs family  There was a wide divergence of needs and wishes expressed by patients regarding whether they would prefer to be on their own or have their partner or family present during discussions about prognosis and EOL issues, suggesting that clarification and negotiation is essential. |
| [5] | Clayton 2005d | 1b. Role and needs family  Several caregivers stated that patients and caregivers have different needs for information concerning prognosis. The specific informational needs of caregivers were often emphasized. For example, caregivers may want information regarding how much time to take off work and other information to plan for future care of the patient. Patients did not provide specific details concerning what type of information would be useful for them versus their caregivers to know.  Some caregivers were concerned that it was unethical to discuss the patient’s prognosis without the patient present. Although many patients said they would be happy for the HP to have a separate discussion with family members regarding their condition, most said they would want to give permission first. In addition, some patients believed they could cope with prognostic information better than their family. Conversely, some patients said it was important to have the support of someone in their family with them during these discussions because of the potential distress that they may feel.  A few caregivers expressed a need to protect the patient from being given distressing news by HPs and emphasized the patient’s right not to know this information. Some patients believed it would be taking away their rights if a physician discussed information about them, including bad news, with their family but not with them. One patient said he believed it was up to the family to decide what information was given to the patient. | 1b. Role and needs family  The current study suggests that terminally ill cancer patients and their caregivers have very different needs for information concerning prognosis and EOL issues. In other cases, although less frequently reported by participants in the current study, the patient may desire more details concerning their prognosis than the caregiver. |
| [6] | Coulourides Kogan 2015 | 1. Patient needs  *Holistic care approach:* The holistic structure of the IPC consults and subsequent related care options was well received by participants. Patients perceived the ability of palliative care to meet the multifaceted physical, emotional, and spiritual health needs of participants as a positive benefit of the holistic care approach.  *Knowledge/information gained:* Hearing the information that was presented in the IPC consults and the subsequent knowledge gained from the discussion) motivated some participants to engage in productive and meaningful decision making around their health status, care, and wishes.  *Hope and enlightenment:* Patient hope or positive expectations were influenced by the information/knowledge and holistic care received during the IPC consults. Additionally, information received from the IPC consult enabled participants to reframe hope from hope of a cure to hope for pain relief and care consistent with their personal goals such as returning home, controlling pain, and spending time with their family members. | 1. Patient needs  *Impact of holistic care on hope*  Two interconnected themes were very closely related to *hope*: holistic care and knowledge/information gained. Patients described how hearing information on the availability of services, hospital resources, and information on holistic nature of palliative care—such as the possibility of symptom control, psychological and spiritual care—made me feel good, enlightened, and motivated. |
| [7] | Curtis 2008 | 1a. Information needs  *Determining “how much information?”:* Patients and families were directly asked how much information they wanted. In response, they said “all the information.” However, in further questioning, a substantial minority of participants made it clear that they did not want explicit information about prognosis such as median survival, estimated life expectancy, or “worst case scenario” and felt that this information could harm them. Throughout the interviews, we found that simply asking patients how much information they want, without exploring their emotions and concerns, did not adequately elicit informational needs.  *Direct versus indirect approach:* Some patients favored a more indirect approach: prognostic information was viewed as a threat to hope and they suggested that a more cautious approach to providing prognostic information might be most helpful. Whereas other patients preferred more direct approaches to prognostic information; they did not report that receiving prognostic information compromised their hopes and they suggested that a more direct and straightforward approach to prognosis would be most helpful. They expressed the need for physicians to communicate fully and explicitly what could happen in the future.  1b: Role and needs family  Some patients differed from their family members in their desire for prognostic information, f.e. with the patient not wanting to know but the partner wanting to know to help prepare the children. Participants who endorsed an indirect approach suggested that physicians should discuss prognosis with the family in situations where they cannot discuss prognosis with the patient. A family member underscored the importance of providing family with information about prognosis, because even if the patient would rather not know, they needed it. | 1a. Information needs  The question “how much information” did not provide patients and families with enough of an opportunity to explain their concerns about receiving explicit prognostic information.  This study suggests that there is important variability in the way that patients with life-limiting diseases, particularly COPD and cancer, approach the interaction of wanting support for hope and wanting explicit prognostic information from their clinicians. Simply asking patients and family how much information they want seems to be an unrevealing approach to understanding individuals’ needs.  1b: Role and needs family  Patients and families differ in their needs and desires for hope and explicit prognostic information. |
| [8] | De Graaff 2010 | 1c. Non-Western patients  **Keeping hope alive**  *Patients and their family:* Patients and their family want care providers not to take away the hope of recovery by talking directly and openly about the negative prognosis. If hope is removed, then the family is afraid that the patient will give up while hope can give him strength to get through this very difficult period.  Some respondents also say that they cannot take away the patients hope for religious reasons: it is for Allah to decide when someone is going to die; life and the possibility of recovery are in Allah’s hands. This is why families ask care providers to be cautious in giving information to the patient. However, often some of the family are informed.  *Different values:* The reaction of doctors to this request of silence from patients’ relatives diverged, with some accepting it while others did not want to take the wishes of the family into account because of their different values. The values of Dutch professionals are contradictory to those of the families of patients with a Turkish or Moroccan background. Dutch professionals are focused on fully informing the patient to reach shared decision making and to realize advanced care planning. Whereas those of families with a Turkish or Moroccan background are centered on keeping patients hope alive, and therefore the family decides how much information can be given to a patient. | 1c. Non-Western patients  **Discussion**  We would recommend that care providers place their own perceptions and practices in perspective, and consider the religious and cultural views of their patients and family members.  **Conclusion**  Taking time and creating opportunities to question mutual expectations wishes and fears can help to avoid frictions and lead to strategies and care interventions acceptable to all parties involved. |
| [9] | De Graaff 2012a | 1c. Non-Western patients  **Multilingual triads**  In only seven of the 33 cases had the patient mastered the Dutch language well enough to communicate independently with the care providers. In the other cases, communication on care and treatment needed to be translated. Relatives often did not consider a professional interpreter to be acceptable, as they feared that the information provided to their beloved sick one would be too direct.  Communication mostly took the form of a triad between HP, the patient and a close relative who spoke Dutch very well, supported the patient and geared the decision-making process to the patients’ own wishes.  **Different expectations of communication**  As far as relatives were concerned, not all subjects were up for discussion. If a doctor for example talked about the diagnosis of cancer with a member of the family (acting as interpreter), he or she generally refused to convey this to the patient.  *Different ideas about the role division in communication:* Communication between care providers and patients of Turkish or Moroccan descent mostly occurred via relatives. Family members acting as interpreters often held a key position in the triad, as they not only translated in the literal sense, but also were able to furnish patient and health care provider with background information. The family often decided when and to what extent the patient should be involved in the conversation.  **Responses of patients and family to conflicting opinions and wishes regarding communication**  When the wishes with regard to communication of healthcare providers differed from those of the family of the patient, the reaction of the family differed. Some grew angry at the care provider, but felt that protests would be to no avail, as they would not be heard, anyway. Other relatives acknowledged that expectations about communication could differ. Some accepted that care providers wished to bring up sensitive subjects that the family would rather avoid. They did feel, however, that care providers should convey painful messages gradually. All relatives indicated that what they found most important was that the care providers recognized them as participants in the communication process. | 1c. Non-Western patients  **Conclusion**  In conclusion, miscommunication around palliative care involves more than different cultural backgrounds and language problems; it may also be caused by the triangular form of communication. Nurses, doctors and other care providers should, therefore, learn how to deal with talking in triads. |
| [10] | De Graaff 2012b | 1c. Non-Western patients  Experiences of Turkish and Moroccan patients, their relatives and their professional care providers differed considerably with regard to communication and decision-making. Four different variants were identified: no agreement between family and care providers rooted in Dutch are providers denial of the existence of the care management group around the patient; communication problems within the family; lack of satisfactory communication and agreement among the professional care providers; and good communication both within the family and with the care providers satisfying all parties.  Identified factors influencing communication were ethnic-cultural differences, language barriers, internal conflicts in families as well as professional teams, professionals who do not accept the family as a care management group, and insufficient acknowledgement of the broker role of the patients social environment by the HCP.  Facilitating factors in communication were tailoring the message to the recipient, and delivering bad news in small doses and in phases. | 1c. Non-Western patients  The concept of care management group rejects the assumption of a simple one-to-one communication between an autonomous patient and an all-knowing professional and emphasizes the broker role of the patients’ social environment.  An important feature of the dynamics in these interactions is that they challenge the principle of autonomy, not only for patients, but also for professionals. This study calls for more sensitivity to the care beliefs and demands of ethnic minorities. |
| [11] | Friedrichsen 2011 | 1a. Information needs  All patients wanted to know the truth, but their definitions of truth varied.  *Absolute truth*: Some patients wanted the factual truth that they are dying and would die within a limited time, containing a clear time limit and definite expectations of what the future would hold.  *Partial truth*: some wanted partial truths and particular facts, but not all of the information. Patients meant that this truth should be positive information that they could benefit from. But hard facts with too detailed information such as having a limited time to live or possibly intolerable symptoms were not included in preferred half-truths. This helped patients maintain some hope.  *Desirable truth*: Some patients made a distinction between the truth that was provided by their physician and what they wanted to hear, their inner desirable truth, the opposite of their situation (i.e. being healthy, cured). This was because they were afraid to discuss this again, not wanting to hear unpleasant information about the future.  The preferred type of truth and corresponding coping strategy impacted on their preferences concerning truth disclosure and physicians’ communication of bad news.  1b. Role and needs family  Some of the patients that wanted only partial truths decided to leave the knowledge about the “real truth” to family members or friends, as they believed that the “real truth” was too much for them. | 1a. Information needs  This study shows that terminally ill patients want to know the truth but the content or definition of this truth might vary. Although patients say, when directly asked, that they want to be told the truth by their doctor, their preferences change when they receive bad news in real life. Therefore, in clinical practice, it could be helpful to ask patients how much and what kind of information they prefer to know about their illness, because the truth is different to different patients. |
| [12] | Hagerty 2005 | 1. Patient needs  *General:* Factor analysis showed that 57% of total variance for preferred *general* doctor behaviors when discussing prognosis was explained by 6 constructs. The two most important constructs of doctor behavior were: 1. Realism and individualized care (providing realistic and direct information tailored to the individual); 22% of total variance. 2. Emotional support (providing information on support services and an openness to discuss patients’ fears and concerns); 12% of total variance. 3. Facilitation of coping with dying (displaying openness to discuss concerns about dying and providing information on palliative care services); 7% of total variance. 4. Provision of information (f.e. ensuring patient understanding); 5% of total variance. 5. Emphasizing all options (f.e. discussing optimistic future scenarios); 5% of total variance. 6. Personal (f.e. share some personal information); 5% of total variance.  *Hope:* Factor analysis showed that 54% of total variance for *hope*-giving behaviors was explained by three factors: 1. Expert/positive/collaborative doctor style (expertise, humor, and inclusion of patient as part of the team); 28% of total variance. 2. Avoidant [negative effect] (avoiding or appearing uncomfortable, giving the prognosis to others first); 14% of total variance. 3. Empathic (expressing one’s own feelings or asking the patient about his or her own reaction to the prognosis); 12% of total variance. |  |
| [13] | Kirk 2004 | 1. Patient needs  **Process**  A number of attributes were identified to be important in communicating information: playing it straight [being honest and direct], showing you care [communicating with compassion and empathy], staying the course [communicating that the patient and family will not be abandoned].  **Content**  *Prognosis:* Participants were distressed when information about prognosis was perceived as vague or inaccurate, was presented along with conflicting or inconsistent information, or was given by someone not perceived to be an expert or directly in charge of the patient. Evasiveness was often perceived as unhelpful.  A subtheme with regard to the prognosis: expert disclosure (the need for prognostic information to be given by the health provider perceived to be an expert).  *Hope:* The second most important content area was the provision of hope and the need for hopeful messages at all stages, described as a possibility for cure or longer life or related to short term visions of the future or continued care or an indication that the health professionals are not giving up. Patients expressed a continuing need for hope even when they knew and accepted that they were in the terminal stages of disease and had a limited life expectancy. To have hope dashed by a rushed or insensitive health carer was experienced extremely negatively.  1a. Information needs  A number of attributes were identified to be important in communicating information: making it clear [conveying information in a way that the patient/family can understand], giving time [providing enough time to the patient and family when discussing information], pacing information [giving information at a rate patients and families can assimilate]. A subtheme with regard to the prognosis: specificity of prognostic information the need for honesty and respect for the level of detail wanted by the patient/family.  1b: Role and needs family  The needs of patients and families were similar but diverged somewhat as the illness progressed. Many patients reported not wanting as much detail about prognosis as they had asked for initially. In early stages families and patients talked to the health carers together. In later stages family members often talked to them alone, often at the patient’s request, and did not confirm the patients’ exact state of knowledge.  Patients and family members did not talk as openly and sheltered each other from knowledge. All reported that they complied with their relatives requests for the amount of information they wanted. Patients focused more on daily living and concerns about managing symptoms; families were more concerned with prognosis and details related to care. Almost all patients wanted to know their prognosis, and family members respected their wish to know or not, although some would have wanted to protect the patient from details regarding prognosis. No family members had requested that the patient was not fully informed. | 1b Role and needs family  Most patients wanted their family member present when they met health carers, although a small number expressed a desire to be the first to know or to control how much or when the family member should be told.  Family members respected the patients wish to know or not, although some would have wanted to protect the patient from details regarding prognosis. No family members had requested that the patient was not fully informed. |
| [14] | Mitchison 2012 | 1a: Information needs  Many of the patients (from all ethnic groups) expressed a preference for oncologists to openly provide all details regarding their prognosis. Reasons for wanting to know were largely pragmatic, as patients felt the information was pertinent to their being able to plan and prepare for the future.  It was more common for Anglo-Australian patients to express not wanting to be told their prognosis, because: statistics are ‘inaccurate’ and unnecessary to know; disclosure would cause unnecessary emotional distress; the patients’ physical condition would deteriorate due to the stress.  A small number of largely Anglo-Australian patients and relatives mentioned that they would prefer prognostic information ‘later on’ when they were ready to receive the information, however, they could still see the benefit of prognostic disclosure to prepare for death.  1b. Role and needs family  Discordance with patient wanting to know their prognosis and the family not wanting prognosis disclosed occurred almost exclusively within migrant sub-samples. In contrast, family-members of Anglo-Australian patients usually supported the patient’s wish to have their prognosis disclosed or not.  Discussing prognosis only in a separate meeting with the relatives was not expressed as preferential by any of the patients. | Anglo-Australian patients often expressed a desire to *not* be told their cancer prognosis. However, some did mention that they would eventually want to know their prognosis.  Usually there was concordance in views between Anglo-Australian family members and patients in communication preferences. |
| [15] | Oosterveld-Vlug 2017 | 1c. Non-Western patients  *Circle of hope:* Muslims regarded it as unacceptable when physicians consciously gave false hope by providing unrealistic information or withholding realistic information (i.e. a circle of hope). However, hope was found to play a different role for Muslims. They pointed out that they always keep their hopes up and rely on their faith in Allah, who is the decider when it comes to life and death.  *Combining Hopeful and Realistic Information:* Muslim participants reported that they prefer physicians to give realistic information rather than unrealistic but hopeful information.  Most participants with a Muslim background preferred that physicians provide realistic information to relatives first. Thereafter, open and explicit communication with the patient does not always occur as family members sometimes choose to not confront the patient with his/her poor prognosis and the fact that they are nearing death.  In addition, Muslim participants detailed how a physician could best communicate that a patient is nearing death: they should inform the patient and/or relatives that they have no curative treatments available, but they should never state that a patient has an “incurable illness,” because physicians are not the ones who decide the question of life and death.  *Shifting of hope:* Muslim participants expressed a preference to involve an imam or Muslim spiritual counselor when the patient was aware of his/her terminal illness and to facilitate end-of-life communication. | 1c Non-Western patients  When realistic information from a medical perspective is introduced in a culturally sensitive way (e.g., not by stating that a patient is incurably ill, but by informing the patient that the physician himself no longer has any available treatment options), hope can be maintained. |
| [16] | Rohde 2019 | 1. Patient needs  The participants’ hopes seemed to change from before they were diagnosed with their incurable disease and through their disease trajectory. Physicians and nurses at the cancer centre conveyed that they would try to delay disease progress and relieve pain and symptoms. Even though they recognised that their cancer was incurable, most participants hoped that they would be among those who could live for years despite a poor prognosis. As the disease progressed, they hoped for good days, not extraordinary things or experiences, and for some participants there seemed to be a change in goals and values.  1a. Information needs  Correct and truthful information about likely future prospects was important for the participants. Preferences varied regarding the amount of information they wanted to receive and at which time point. Some participants wanted a total overview of their disease and prognosis from the start, some wanted a smaller amount of information at that time, while others wanted their body to tell them how their disease was progressing. Some participants found vague information about likely prospects confusing. | 1. Patient needs  The realistic hope for most of our participants was that something could be done to relieve their symptoms and potentially to postpone death, and to enable them to lead ordinary everyday lives and have the possibility of spending time with family and friends. Therefore, the participants emphasised the importance of HCPs including hope in their communications of disease, prognosis and life expectancy throughout the disease trajectory. |
| [17] | Walczak 2013 | 1. Patient needs  *Skills, understandings and relationship elements:* Patients identified important doctor skills when discussing prognosis. These included maintaining a calm and open manner in all contacts with the patient, treating the patient as an individual and being sensitive to their individual needs. Participants also indicated that control of discussions should be actively given to the patient, but that the doctor should also take the initiative to raise complex or difficult topics such as prognosis and end-of-life issues. In discussing these issues and responding to patients’ questions, participants felt that doctors should be honest, acknowledge and explain uncertainty where it exists, and relate the stories of other patients to foster hope and illustrate uncertainty.  Also contributing to adjustment and acceptance was a good doctor/patient relationship that was characterised by patients as a feeling of comfort and trust in their doctor.  Finally, a clear and explicit agreement and permission from both parties to discuss these complex and difficult topics was highlighted as an important step towards readiness to discuss end-of-life issues. Patients recognised that not only was it important for the doctor to invite discussion of prognosis and end-of-life issues, but also that the patient needed to give their doctor permission to raise these issues as well. | 1a. Role and needs family  Participants in this study also noted the important role of family in shaping patients acceptance and readiness to discuss prognosis and end-of-life issues. |
