# Supplementary material for: Discussing personalized prognosis in amyotrophic lateral sclerosis: development of a communication guide
Source: BMC Neurol. 2020 Dec 14;20:446. doi: 10.1186/s12883-020-02004-8 (PMC7734773; doi:10.1186/s12883-020-02004-8)
Supplement: Supplementary file 5 — Additional file 5. Table 3. Synthesis of findings. [file 12883_2020_2004_MOESM5_ESM.docx]

Table 3. Synthesis of findings

| **Topic** | **Theme** | **Synthesis of findings** | **Summary of evidence** |
| --- | --- | --- | --- |
| 1. Patient needs | Honest communication with empathy | Overall, studies showed that patients desire prognostic information to be disclosed in an open, honest, and straightforward manner without being blunt and with empathy [1, 3, 4, 13, 14, 17]. | - Most patients wanted prognostic information to be disclosed in a straightforward, honest manner, if desired [1].  - Several patients said that it is important to be honest when discussing the future, without being too blunt. None indicated that they did not want their HP to be honest [3].  Patients said that it is vital for the HP to show compassion and respect, some spoke of the devastating effect of having bad news broken when the doctor did not show any signs of compassion [4].  - Patients prefer realistic information, emotional support and the physician not being avoidant [12].  - When discussing prognosis patients desire honesty and directness, but with empathy [13].  - Many patients preferred to openly be provided with all details on their prognosis [14].  - Patients prefer their doctor to be honest and acknowledge uncertainty where it exists when discussing their prognosis [17]. |
|  | Hope- and hope-giving | Hope has been reported as important in prognostic communication to help counterbalance patients’ need for the truth. Prognostic discussion can help patients redefine hope for a cure to hope consistent with personal goals like being with family or being well cared for [1, 3, 6, 13, 16].  Hope can be supported in a number of ways: emphasizing the holistic nature of palliative care [3, 6, 12, 16]; reassurance of non-abandonment [3, 6]. | - Hope was seen as a vital part of prognostic discussions [1].  - Patients stated that hope can be nurtured by reassuring patients that pain and other symptoms can be controlled, emotional support, knowing that they will not be abandoned, and information about equipment and resources. But patients also underscored that it is important for HPs not to give false hope but any positive aspects should be emphasized. A few patients spoke of the hope of beating the odds and being on the tail of the survival curve [3].  - Patients perceived the holistic approach of palliative care aimed at physical, emotional, and spiritual health needs of participants as hope-giving. This information also helped them redefine their hope [6].  - Patients prefer to hear information about palliative care while discussing prognosis [12].  - Patients expressed a continuing need for hope [13].  - Patients’ redefined their hope in line with their new goals, like spending time with their families. Emphasis on care options to delay death or relieve symptoms helped support hope [16]. |
|  | Trusted expert physician | Patients prefer the prognosis to be communicated by a physician they know, trust, feel comfortable with, and consider an expert [1, 4, 12, 13, 17]. | - Patients wanted to hear their prognosis from their oncologist whom they knew and trusted [1].  - Patients strongly emphasized the importance of being comfortable with their HP when discussing prognosis [4].  - Patients preferred a physician they considered to be an expert to discuss their prognosis with them [12].  - Patients desire their prognosis to be disclosed by someone they perceive to be an expert [13].  - Patients wanted a feeling of comfort and trust in their doctor when discussing prognosis [17]. |
|  | Physician initiative | Studies show that the physician can take the initiative to broach the subject of life expectancy, as long as patients are given the option to say no [4, 17]. | - Provided the patient is given the option not to hear the prognosis and the topic is broached in a sensitive manner, most participants felt that it was appropriate and important for the doctor to make this an accessible topic, because the patient might find it difficult to raise it themselves [4].  - Patients felt that the doctor should take the initiative in raising the topic of prognosis, but should leave the patient in control whether to discuss it or not [17]. |
|  | Respecting cultural values | Tailoring information also encompasses respecting the cultural values of patients and their families by 1) exploring differences in values between healthcare professionals and the patient and their family, and how these might lead to different communication needs, 2) acceptance of a more central role of the family in the communication and care process [8–10, 15]. | - It is recommended that HP’s their own perceptions and practices in perspective, and consider the religious and cultural views of their patients and family members when discussing prognosis with Muslim patients [8].  Miscommunication around palliative care involves more than different cultural backgrounds and language problems; it may also be caused by the triangular form of communication between HP’s , Muslim patients and their relatives [9].  The outcome is not a simple one-to-one communication between an autonomous patient and an all-knowing professional, the social environment of the Muslim patients plays an important role. This study calls for more sensitivity to the care beliefs and demands of ethnic minorities [10].  - When realistic information from a medical perspective is introduced in a culturally sensitive way (e.g., not by stating that a patient is incurably ill, but by informing the patient that the physician himself no longer has any available treatment options), hope can be maintained [15]. |
|  | Spiritual support | Spiritual support, if desired by the patient, can help support a patient’s hope [6]. If needed, a priest [4], imam [15], or spiritual counsellor can help facilitate communication on life expectancy and help patients and their family make the transition from hope for a cure to hope for a good death. | - One carer said her husband wanted their priest and not the doctor to deliver any bad news and had requested this be documented in the medical record [4].  - Spiritual support was considered a part of holistic, palliative care that supported hope [6].  - Muslim participants expressed a preference to involve an imam or Muslim spiritual counselor when the patient was aware of his/her terminal illness and to facilitate end-of-life communication [15]. |
| 1a. Information needs | Tailored information | Information needs of patients differ and prognostic discussion should be tailored to each patient’s needs, some want more explicit prognostic information and time frames whereas others desire a more general indication [1, 2, 4, 7, 11–14, 16].  Simply asking how much patients want to know without explaining what kind of information is available and exploring their emotions and concerns, might not sufficiently elicit informational needs [1, 7, 11].  Statistics and time frames should be used cautiously, not all patients wanted to hear them because they feared they could potentially cause distress and threaten their hope (1,14). Additionally, patients emphasized it should be explained that statistics are inaccurate and apply to groups rather than individuals (2,14). Finally, some patients preferred for positive aspects to be emphasized and obtained hope from good news stories of patients who lived longer than average [1–3, 17]. | - Patients felt that doctors should explore what information they want and how such information might most usefully be imparted to them. The end result of such a discussion may be far from a survival curve. Statistics, especially a time frame, were seen as potentially hope destroying and wanted to hear “good news” stories [1].  - Not all patients wanted detailed information about their life expectancy. Some wanted a survival time frame, but many others wanted only a general indication. Those patients who wanted to be given a time frame mostly wanted to know how long the average person with their condition would live and/or be given a rough range. A few spoke of the hope of beating the odds and being on the tail and of the survival curve, and they wanted positive aspects to be emphasized. Patients also said that it is important to explain that statistics apply to a group so they can only be used as a guide. Patients and carers wanted their HPs to highlight that every person is an individual and that people’s experiences are different even with the same disease [2, 4].  - Patients and family responded that they wanted “all the information.” However, in further questioning, a substantial minority of participants made it clear that they did not want explicit information about prognosis such as a median survival or estimated life expectancy. Simply asking patients how much information they want, without exploring their emotions and concerns, did not adequately elicit informational needs. Some patients favored a more indirect approach, whereas other patients preferred more direct, full and explicit approaches to prognostic information [7].  - All patients wanted to know the truth, but their definitions of truth varied. Some wanted a clear time limit, but others were not interested [11].  - Patients preferred information tailored to the individual while discussing prognosis [12].  - Patients wanted information to be communicated in a way that they can understand, at a rate that they can assimilate and adjusted to the level of detail they want to know [13].  - Many patients preferred open discussion of all details regarding their prognosis. However, there were also many patients who did not want to be told their prognosis because statistics are inaccurate and fear of distress; a number of them might want information later on [14].  - Preferences varied regarding the amount of information patients wanted to receive, some wanted a total overview of their prognosis from the start, whereas others wanted a smaller amount of information at that time. Uncertainty should be acknowledged, but stories of other patients could also foster hope [17]. |
| 1b. Role and needs family | Family for support | The presence of family can provide the patient emotional support during prognostic discussion, but who if anyone should be present should be negotiated in advance [4, 5, 13, 17]. | - Patients said that it is vital that the doctor ensure that support is present when discussing prognosis. Most patients wanted someone from their immediate family present, but felt that it was important to negotiate who should be present when bad news was given [4, 5].  - Most patients wanted family present, some did prefer this to be negotiated with them first. Almost all patients wanted to know their prognosis, and family members respected their wish to know or not, although some would have wanted to protect the patient from details regarding prognosis. No family members had requested that the patient was not fully informed [13].  - Participants considered the role of the family as important for readiness of patients to discuss prognosis [17]. |
|  | Diverging information needs | Information needs can diverge between patients and their families [2, 5, 7, 11, 13], especially their partners or informal caregivers who might feel a stronger need to plan for the future and care needs [2, 5, 13]. Some patients might not be interested in their prognosis, but this can often be discussed with their family if they desire to know and the patient has given permission [5, 7, 11].  Although family members might want to protect patients from hearing bad news about their prognosis, they respected the patients’ right and wish to know [13, 14]. | - Some carers said they have a different reason for needing a time frame than the patient: for example, knowing how much time to take off work and whether to call other family members to share the care-giving burden [2, 5]. Many patients said they would be happy for the HP to have a separate discussion with family members regarding their condition, most said they would want to give permission first [5].  - Some patients differed from their family members in their desire for prognostic information, f.e. with the patient not wanting to know but the partner wanting to know. In these situations participants suggested that physicians should discuss prognosis with the family [7].  - Those patients that did not want information about a time frame left knowledge about this to their family [11].  - The needs of patients and families were similar but diverged somewhat as the illness progressed. Patients focused more on daily living and concerns about managing symptoms; families were more concerned with prognosis and details related to care [13].  - There was no discordance between patients and their families regarding disclosure of prognosis as families supported patient’s wish to know or not [14]. |
| 1c. Non-western patients in the Netherlands | Conspiracy of silence | Studies in the Netherlands showed that families of non-western patients, specifically Muslim patients, can prefer to act as intermediate between patient and physician when prognosis is discussed. The family often maintains a conspiracy of silence towards the patient on the topic in order to protect the hope of the patient and because of religious taboo [8, 9, 15]. This can create tensions when juxtaposed with Dutch healthcare provider’s values aimed at communicating directly with the patient and fully informing them [8]. | - Patients and their family want HP’s not to take away hope by talking directly and openly about the negative prognosis. Hope also cannot be taken away for religious reasons, since Allah decides whether someone lives or dies. Therefore families ask HP’s to be cautious in giving information to the patient, but inform the family. This can be difficult for HP’s since it often clashes with their values on fully informing the patient and supporting informed decision-making [8].  Due to language difficulties communication often takes the form of a triad, between HP, patient, and a relative who translates and gears decision-making to the patients’ own wishes. Relatives often did not consider a professional interpreter to be acceptable, as they feared that the information provided to the patient would be too direct. Difficult topics, like prognosis, would not always be translated and communicated to the patient by relatives who decided when and to what extent the patient should be involved in the conversation. Relatives felt that care providers should convey painful messages gradually and recognize them as participants in the communication process [9].  - Muslims regarded it as unacceptable when physicians consciously gave false hope by providing unrealistic information or withholding realistic information, they prefer realistic information. However, they also preferred that physicians provide realistic information to relatives first. Thereafter, open and explicit communication with the patient does not always occur as family members sometimes choose to not confront the patient with his/her poor prognosis and the fact that they are nearing death.  Hope was found to play a different role for Muslims, who always have to keep their hopes up and rely on their faith in Allah, who is the decider when it comes to life and death [15]. |
| 1d. Cognitive impairment and frontotemporal dementia | - | - | No evidence was found. |
