# Supplementary material for: Discussing personalized prognosis in amyotrophic lateral sclerosis: development of a communication guide
Source: BMC Neurol. 2020 Dec 14;20:446. doi: 10.1186/s12883-020-02004-8 (PMC7734773; doi:10.1186/s12883-020-02004-8)
Supplement: Supplementary file 6 — Additional file 6. Full list of included studies. [file 12883_2020_2004_MOESM6_ESM.docx]

**Full list of included studies**

1. Butow P, Dowsett S, Hagerty R, Tattersall M. Communicating prognosis to patients with metastatic disease: what do they really want to know? Support Care Cancer. 2002;10:161–8. doi:10.1007/s005200100290.

2. Clayton JM, Butow PN, Arnold RM, Tattersall MHN. Discussing life expectancy with terminally ill cancer patients and their carers: a qualitative study. Support Care Cancer. 2005;13:733–42. doi:10.1007/s00520-005-0789-4.

3. Clayton JM, Butow PN, Arnold RM, Tattersall MHNN. Fostering coping and nurturing hope when discussing the future with terminally ill cancer patients and their caregivers. Cancer. 2005;103:1965–75. doi:10.1002/cncr.21011.

4. Clayton JM, Butow PN, Tattersall MHN. When and How to Initiate Discussion About Prognosis and End-of-Life Issues with Terminally Ill Patients. J Pain Symptom Manage. 2005;30:132–44. doi:10.1016/j.jpainsymman.2005.02.014.

5. Clayton JM, Butow PN, Tattersall MHN. The needs of terminally ill cancer patients versus those of caregivers for information regarding prognosis and end-of-life issues. Cancer. 2005;103:1957–64. doi:10.1002/cncr.21010.

6. Coulourides Kogan A, Penido M, Enguidanos S. Does Disclosure of Terminal Prognosis Mean Losing Hope? Insights from Exploring Patient Perspectives on Their Experience of Palliative Care Consultations. J Palliat Med. 2015;18:1019–25. doi:10.1089/jpm.2015.0038.

7. Curtis JR, Engelberg R, Young JP, Vig LK, Reinke LF, Wenrich MD, et al. An Approach to Understanding the Interaction of Hope and Desire for Explicit Prognostic Information among Individuals with Severe Chronic Obstructive Pulmonary Disease or Advanced Cancer. J Palliat Med. 2008;11:610–20. doi:10.1089/jpm.2007.0209.

8. de Graaff FM, Francke AL, van den Muijsenbergh ME, van der Geest S. “Palliative care”: a contradiction in terms? A qualitative study of cancer patients with a Turkish or Moroccan background, their relatives and care providers. BMC Palliat Care. 2010;9:19. doi:10.1186/1472-684X-9-19.

9. de Graaff FM, Francke AL, Van den Muijsenbergh ME, van der Geest S. Talking in triads: communication with Turkish and Moroccan immigrants in the palliative phase of cancer. J Clin Nurs. 2012;21:3143–52. doi:10.1111/j.1365-2702.2012.04289.x.

10. de Graaff FM, Francke AL, van den Muijsenbergh METC, van der Geest S. Understanding and improving communication and decision-making in palliative care for Turkish and Moroccan immigrants: a multiperspective study. Ethn Health. 2012;17:363–84. doi:10.1080/13557858.2011.645152.

11. Friedrichsen M, Lindholm A, Milberg A. Experiences of truth disclosure in terminally ill cancer patients in palliative home care. Palliat Support Care. 2011;9:173–80. doi:10.1017/S1478951511000046.

12. Hagerty RG, Butow PN, Ellis PM, Lobb EA, Pendlebury SC, Leighl N, et al. Communicating With Realism and Hope: Incurable Cancer Patients’ Views on the Disclosure of Prognosis. J Clin Oncol. 2005;23:1278–88. doi:10.1200/JCO.2005.11.138.

13. Kirk P, Kirk I, Kristjanson LJ. What do patients receiving palliative care for cancer and their families want to be told? A Canadian and Australian qualitative study. BMJ. 2004;328:1343. doi:10.1136/bmj.38103.423576.55.

14. Mitchison D, Butow P, Sze M, Aldridge L, Hui R, Vardy J, et al. Prognostic communication preferences of migrant patients and their relatives. Psychooncology. 2012;21:496–504. doi:10.1002/pon.1923.

15. Oosterveld-Vlug MG, Francke AL, Pasman HRW, Onwuteaka-Philipsen BD. How should realism and hope be combined in physician–patient communication at the end of life? An online focus-group study among participants with and without a Muslim background. Palliat Support Care. 2017;15:359–68. doi:10.1017/S1478951516000833.

16. Rohde G, Söderhamn U, Vistad I. Reflections on communication of disease prognosis and life expectancy by patients with colorectal cancer undergoing palliative care: a qualitative study. BMJ Open. 2019;9:e023463. doi:10.1136/bmjopen-2018-023463.

17. Walczak A, Butow PN, Davidson PM, Bellemore FA, Tattersall MHN, Clayton JM, et al. Patient perspectives regarding communication about prognosis and end-of-life issues: How can it be optimised? Patient Educ Couns. 2013;90:307–14. doi:10.1016/j.pec.2011.08.009.
